# Geographical location influence ‘*Cabernet Franc*’ fruit quality in Shandong province

**DOI:** 10.1038/s41598-023-50140-1

**Published:** 2024-01-29

**Authors:** Chaoping Wang, Xueqin Chen, Yanhua Ren, Xuxian Xuan, Tariq Pervaiz, Lingfei Shangguan, Jinggui Fang

**Affiliations:** 1 College of Horticulture, Nanjing Agricultural University, Nanjing, Jiangsu Province China; 2 Shandong Academy of Grape, Jinan, Shandong Province China

**Keywords:** Plant physiology, Plant molecular biology, Environmental impact

## Abstract

Grape quality is a key factor in determining wine quality, and it depends not only on management skills, but also on the geographic location of the producing area. In China, Shandong is the province with the largest wine production, and ‘*Cabernet Franc*’ is widely planted. This study evaluated the ‘*Cabernet Franc*’ fruit quality in relation to geographical conditions in five ‘*Cabernet Franc*’ producing districts of Shandong province, China, including Dezhou Aodeman Winery (DZ), Tai’an Zhongqingsongshi Winery (TA), Penglai Longhu Winery (PL), Rushan Taiyihu Winery (RS), and Rizhao Taiyangcheng Winery (RZ). At the time of veraison and maturity, fruit was harvested from five areas, and compared for cosmetic and internal fruit quality. The soluble sugar content in the Rizhao area was rich, and the weight and volume of single fruit were relatively large. The titratable acid of the berries in Tai'an area was high. RNA-seq analysis showed that the number of genes in the véraison stage was 19,571–20,750, and the number of genes in the mature stage was 19,176–20,735. The analysis found that areas with multiple high-quality characteristics tended to have more DEGs (differential expressed genes). And the DEGs in different areas were mainly distributed on chromosome 7, and at least on chromosome 15. DEGs in 5 areas were enriched on 855 GO terms and 116 KEGG pathways during berries development. Among them, it was found that the up/down-regulation of DEGs was related to the formation of berry quality, which helps to explain the impact of environment on grape quality components. In summary, this study is helpful to understand the influence of cultivation location on the quality of '*Cabernet Franc*' in different production areas in Shandong province, and further provide a reference for the production of high-quality wine grapes and winemaking.

## Introduction

Shandong province is the largest wine produceing area in China, located on the eastern coast of China and the lower reaches of the yellow river. Its territory includes the peninsula and the interior. The central part of the territory is protruding, the southwest and northwest are low-lying and flat, and the east is gently undulating, forming a general terrain with mountains and hills as the skeleton and plains and basins interlaced. The landforms in the territory are complex and can be roughly divided into basic landform types such as plains, terraces, hills, and mountains. Shandong province has a temperate monsoon climate with concentrated rainfall and four distinct seasons, cold and dry in winter and wet and rainy in summer, providing conditions for the cultivation of wine grapes. Shandong province is a major wine-producing area in China, and its wine production ranks first in China. As early as 1892, Shandong yantai changyu company began to introduce grape varieties from abroad and promote their cultivation.

The quality of grape is very important to wine processing, and varies from area to area^1^. In the past, researchers have found that soil^2,3^, moisture^4^, sunlight^5^, solar radiation^6^ and temperature^7,8^ directly affect grape quality during fruit ripening. Previous studies have found that lack of water increases sugar and anthocyanin content in berries^9,10^. Altitude also correlates with grape quality. At low altitudes, grapes have higher tartaric acid and citric acid; higher altitudes have higher malic acid, succinic acid and anthocyanin in the peel^11^. The ‘*Cabernet Franc*’ grape is one of the commonly used varieties of red wine, and it is very popular in China. ‘*Cabernet Franc*’ grapes have strong cold resistance, high sugar content and unique flavor. The wines made with it are fruity and high-quality.

With the rapid development of viticulture and wine industry in China, more and more attention has been paid to the growth of grapes and the formation of fruit quality. Wine grape quality mainly includes sugar and acid content, anthocyanin content. The sugar in grape is one of the important factors that affect the quality of grapes. It was fermented by yeast to produce alcohol. The main sugars components in grapes are glucose and fructose, and most grape varieties contain only a small amount of sucrose^12^. Malic acid, citric acid, and tartaric acid are the main reasons for the sour taste of grape juice^13^. Compared with sugar, the content of organic acids in grapes although lower, but organic acids have an important impact on grape quality. Anthocyanin in grape peel is one of the important criteria for evaluating the quality of wine grapes, it is a flavonoid compound that determines the color of grapes. The influence of geographical conditions on fruit quality is an important part of this study. The geographical location of grape planting is related to the quality of grapes, because the geographical location of planting is directly related to environmental factors such as temperature, humidity, sunlight, altitude, etc. that affect grape ripening. The characters of the same variety in different areas are different and have obvious regional.

Environmental factors such as temperature, light, and cold associated with viticulture have been extensively studied. However, little is known about the relationship between geographical location and grape quality, and there are few systematic studies on this aspect. Understanding the quality of grapes in each area is the core of quality assessment, and these studies are particularly important for the development of wine-producing areas. In order to explore the characteristics of ‘*Cabernet Franc*’ in different areas, we analyzed and compared the quality characteristics of ‘*Cabernet Franc*’ in five areas of Shandong province. Moreover, we used RNA-seq to identify differentially expressed genes (DEGs) in berry véraison and maturity stages in five areas, and screened out GO pathways and KEGG pathways related to quality, providing supplements for the differences in grape traits between five areas. It lays the foundation for further revealing the molecular mechanism of how geographical regulate grape quality, and at the same time, provides a reference for the production of better quality wine grapes.

## Materials and methods

### Plant materials

**Figure 1:**
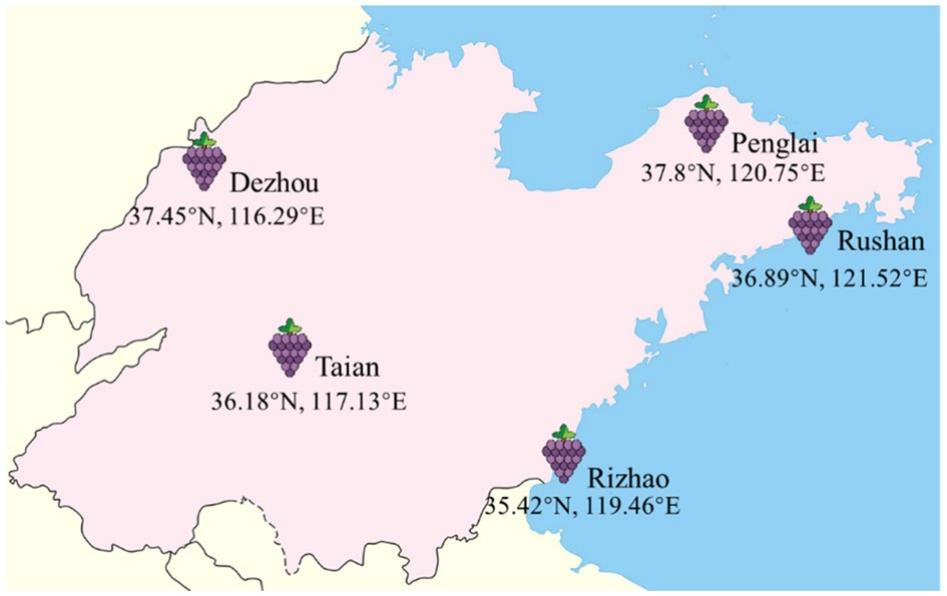
Sample collection distribution map.

‘*Cabernet Franc*’ was selected as the measure material, the samples were collected at véraison stage (50% of the fruit peel begin to change color, 60 days after flowering) (V) and maturity stage (fruit peel coloring was completed and TSS or titratable acidity tended to be stable) (M) from the vineyard of Shandong province, including Dezhou Aodeman Winery (DZ), Tai’an Zhongqingsongshi Winery (TA), Penglai Longhu Winery (PL), Rushan Taiyihu Winery (RS), and Rizhao Taiyangcheng Winery (RZ) (Fig. 1), marked as DZV, DZM, TAV, TAM, PLV, PLM, RSV, RSM, RZV, and RZM. Appropriate permission has been obtained for collection of plant material. Experimental research and field studies on plants, including the collection of plant material, comply with relevant institutional, national, and international guidelines and legislation. The climate profile of the test winery is shown in Table 1. All plant material was grown east–west and managed using the same viticultural practices (5-year-old grape, pruning system, fertilization and crown management) and harvested in 2020. The samples were immediately frozen in liquid nitrogen individually. All samples were stored at – 80 °C for subsequent analysis.

**Table 1:** Climatic characteristics of different wineries.

Name of the winery	Located in the area	Climate	Soil	Remark
Tai’an Zhongqingsongshi Winery (TA)	Mountainous and hilly areas in central shandong	Warm temperate continental semi-humid monsoon climate	Loam	Light and heat are synchronized, rain and heat are at the same time, the annual average precipitation is less, and the annual average sunshine time is 2313.2 h, 193 m above sea level
Penglai Longhu Winery (PL)	The northernmost point of jiaodong peninsula	Temperate monsoon continental climate	Mainly brown loam soil, high gravel content, good water permeability	Adequate sunshine, abundant heat and moderate rainfall, 68 m above sea level
Rushan Taiyihu Winery (RS)	Southeastern tip of jiaodong peninsula	Temperate monsoon continental climate	Mainly brown loam soil, high gravel content, good water permeability	Adequate sunshine, abundant heat and moderate rainfall, 27 m above sea level
Dezhou Aodeman Winery (DZ)	Plains in the east and west of the mountain	Temperate monsoon climate	Mainly sandy loam and light loam	Abundant heat resources, rain and heat in the same season, 25 m above sea level
Rizhao Taiyangcheng Winery (RZ)	Rainy areas in southern shandong	Mild and humid continental climate	Mainly shajiang black soil and fluvo-aquic soil	Sufficient rainfall, with an average annual rainfall of more than 800 mm, and sufficient sunshine, 146 m above sea level

### Physiological and biochemical index determination

Fruit longitudinal diameter and transverse diameter were determined by vernier caliper, grain weight determined by electronic balance. Total soluble solid (TSS) determined by hand-held refractometer (PAL-1, Japan). Titratable acid (TA) determined using sodium hydroxide solution titration. Soluble sugar content determined using anthrone-sulfuric acid method, after obtaining the grape pulp extract, add anthrone ethyl acetate reagent and concentrated sulfuric acid, immediately put the tube into the boiling water bath after fully shaking, keep it warm for 1 min, take it out, cool it down to room temperature naturally, and the absorbance of samples at 765 nm with multi-detection microplate reader (CYTATION3, BioTek, Winooski, VT, USA).

The anthocyanin content was determined using pH differential method. The samples to be tested were ground with liquid nitrogen and freeze-dried. Weigh 50 mg of lyophilized powder and extract with 10 mL of 0.1% hydrochloric acid–methanol (V/V) solution at 4 °C for 12 h, during which time vortex 2–3 times. Finally, the extract was centrifuged at 12,000 rpm for 20 min to obtain a supernatant. Use the buffer solution of pH 1.0 (25 mmol/L KCl, pH adjusted to 1.0 with HCl) and the buffer solution of pH 4.5 (400 mmol/L NaAc, pH adjusted to 4.5 with HCL) to extract according to the same multiple dilute, and measure the absorbance of the two diluted solutions at 520 nm and 700 nm.

Sugar components mainly glucose and fructose were determined by HPLC (Agilent, Santa Clara, CA, USA, Waters, Milford, MA, USA) according to a method previously reported with some modification^14^. Three replicates were measured by HPLC. In a 2 mL Eppendorf tube, 0.50 g of berries of each sample were taken and mixed with 1.5 mL 80% ethanol and put in a water bath at 80 °C for 30 min. The samples were centrifuged at 12,000 rpm for 10 min, and then the extract was filtered through a 0.22 μm water filter for injection. The chromatographic conditions for the determination were as follows: Prevail Carbohydrate ES 5μ column (100 mm × 4.6 mm, 5 μm); mobile phase: acetonitrile: water (80%: 20%); column temperature: 50 °C; flow rate: 1.0 mL/min; injection volume: 20 μL. Sugar standards (fructose and glucose) were purchased from Shanghai Macklin Biochemical Technology Co., Ltd. (Shanghai, China).

Acid components were determined by HPLC (Agilent, USA, Waters) according to a method previously reported with some modifications^14^. Three replicates were measured by HPLC. The aforementioned extraction method is identical to the extraction of sugar. The chromatographic conditions for the determination were as follows: column. Discovery C18 column (25 cm × 4.6 mm, 5 m); mobile phase: 50 mM K2HPO4 solution (pH adjusted to 2.4 with phosphoric acid); column temperature 30 °C; flow rate 0.5 mL/min; injection volume 20 μL, detection wavelength was 210 nm. Organic acid standards (tartaric acid, malic acid, and citric acid) were purchased from Shanghai Macklin Biochemical Technology Co., Ltd. (Shanghai, China).

### Transcriptomic analysis

Total RNA was extracted using the cetyltrimethylammonium bromide (CTAB) method. Library preparation and transcriptome sequencing was completed by the Beijing Novogene Technology Corporation (Beijing, China). Two biological replicates from each sample were used for transcriptome sequencing. Transcriptome data of ‘*Cabernet Franc*’ from multiple areas were registered in NCBI and the accession number of RNA-seq data is PRJNA932956. Raw sequences were filtered to remove the adaptor sequence, low-quality reads, and short reads. The resulting sets of high-quality clean reads were used for transcriptome analysis. High-quality clean reads were mapped to the grape reference genome (Vitis vinifera cultivar: *Muscat Hamburg* (wine grape)) (https://www.ncbi.nlm.nih.gov/bioproject/671671, accessed on 18 december 2020) to obtain uni-genes using Hisat2 v2.0.5^15^. The expressed values of all genes were calculated and normalized according to fragments per kilobase of transcript per million mapped reads (FPKM). Differentially expressed genes (DEGs) analysis was performed using the DESeq R package (1.18.0) (Benjamini and Hochberg, 1995). the GOseq R software package and KOBAS software were used to analyze the GO enrichment and KEGG pathway^16–18^.

### Statistical analysis

Samples were performed with at least three replications and analyzed using SPSS 17.0 (SPSS Inc, Chicago, ILL, USA), TBtools v1.072, and Origin Pro 9 (Origin Inc., Northampton, MA, USA). MapMan (version 3.6.0RC1, Berlin, Germany) was used to present the differences in the expression of genes involved in various functional modules. Excel 2019 is used for statistics and analysis of data.

## Results

### Characteristics of mature ‘*Cabernet Franc*’ fruits collected from different producing areas

**Figure 2:**
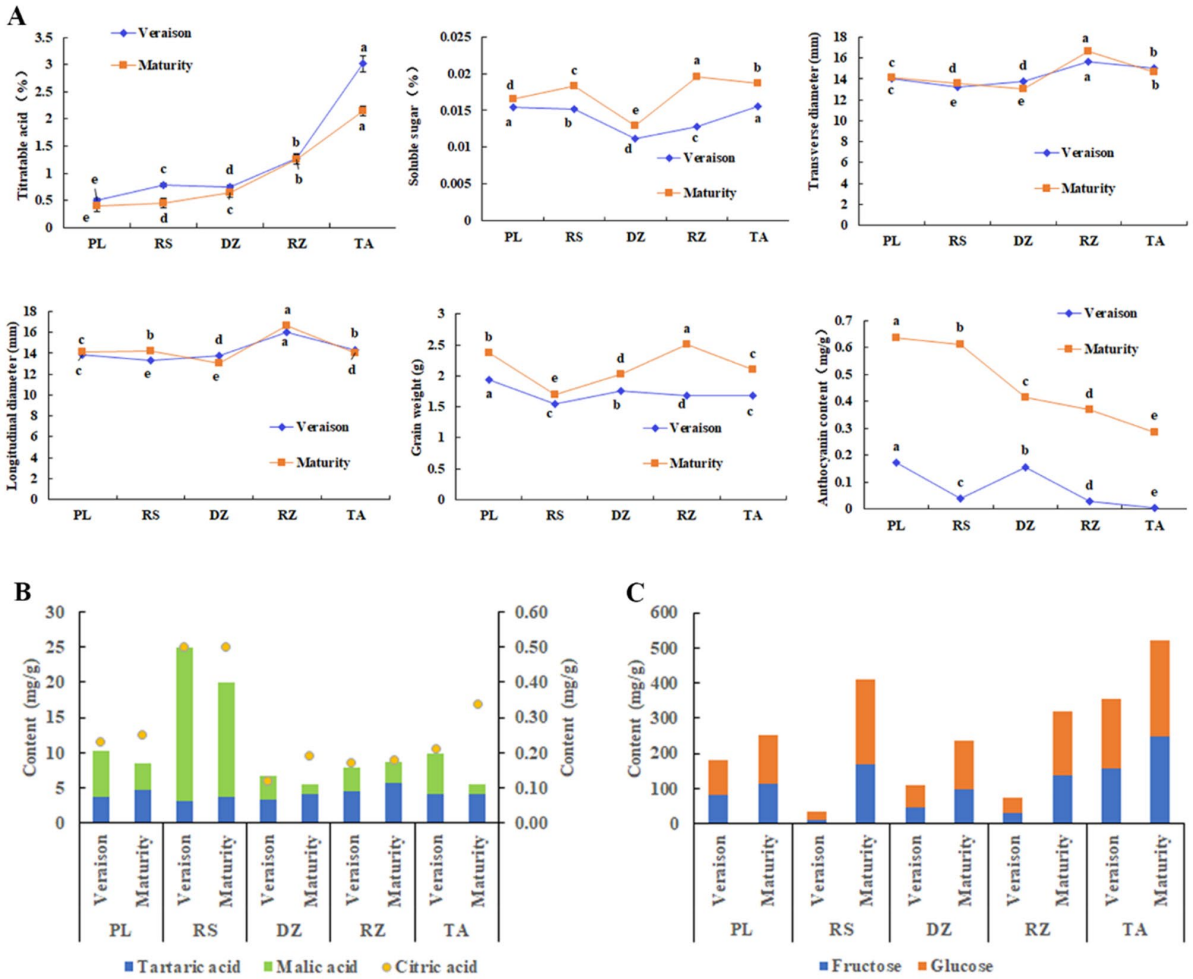
(**A**) Agronomic characters of véraison stage and maturity of ‘*Cabernet Franc*’ in five different area. Penglai (PL)/Rushan (RS)/Dezhou (DZ)/Rizhao (RZ)/Tai’an (TA). Different lowercase letters indicate that through Duncan's test, there were significant differences in fruit quality traits between the 5 ecological area (*p* ≤ 0.05); (**B**) ‘*Cabernet Franc*’ fruit acid component content. Blue represents tartaric acid; green represents malic acid; orange represents citric acid. Since the citric acid content was much smaller than the tartaric acid and malic acid content, the citric acid content was represented by a scatter plot alone, corresponding to the Y-axis on the right; (**C**) The sugar content of '*Cabernet Franc*' sugar components.

The analysis showed that mature ‘*Cabernet Franc*’ fruits differed in agronomic traits in different areas (Fig. 2A). The soluble sugar content of the five wineries ranged from 0.013 to 0.020%, of which the Rizhao was richer; the titratable acid ranged from 0.39 to 2.15%, of which the Tai'an was more abundant; the range of anthocyanin content was 0.29–0.63 g, among which, the content of anthocyanins in Penglai was relatively rich; the range of single fruit weight was 1.7–2.51 g, and the single fruit weight in Rizhao was larger; the range of longitudinal and transverse diameters was 13.1–16.7 mm, among them, the longitudinal and transverse diameters of the Rizhao were longer.

Other studies found that the content of sugar and acid components varied in different areas (Fig. 2B,C). In terms of sugar components, ‘*Cabernet Franc*’ has similar content of glucose and fructose. Among them, Tai'an was rich in glucose and fructose, with 248.4 mg/g and 273.32 mg/g, respectively; the glucose and fructose contents in Dezhou were lower, only 137.14 mg/g and 98.51 mg/g, respectively. In terms of acid components, the main components of acid were varied in different areas. Penglai, Dezhou, Rizhao, and Tai'an were all dominated by tartaric acid, but Rushan was dominated by malic acid. Among them, the content of tartaric acid in Rizhao was the most abundant, with 5.77 mg/g; the content of malic acid and citric acid in Rushan was the most abundant, with 16.32 mg/g and 0.5 mg/g, respectively.

### The variation of the traits from véraison to mature stage

During the véraison period, the characteristics of ‘*Cabernet Franc*’ varies from different areas. Basically, areas with excellent agronomic traits at maturity also have excellent agronomic traits at the véraison stage. The characteristics of '*Cabernet Franc*' varied differently in 5 areas from véraison to maturity (Fig. 2A). Among them, the longitudinal diameter, transverse diameter and titratable acid of the fruits changed little, while anthocyanins and single fruit weight changed greatly. The anthocyanin of Rushan changed greatly, increased by 93%; the soluble sugar and single grain weight of Rizhao changed greatly, increased by 34.6% and 9.4% respectively.

### Transcriptome analysis of the ‘*Cabernet Franc*’ fruits collected at véraison and mature stages in different areas

In order to determine the expression genes in ‘*Cabernet Franc*’ fruit collected at maturity and véraison, RNA-seq was performed on the véraison and ripening stages of ‘*Cabernet Franc*’ fruits in five area of Shandong province: Penglai, Rizhao, Tai’an, Rushan, and Dezhou. A total of 125.11 Gb of raw data was obtained (Table 2). Clean reads accounted for 93–98% of raw reads, GC content was 46.74–48.19%, and Q30 bases accounted for more than 93.37%. The sequencing quality was good, and the sequencing data could be used for further analyze. The clean data after quality control was compared to the reference genome, more than 75% of the reads of all samples were corresponded to the reference genome of fruits, and more than 71% of the reads could be corresponded to the unique position of the reference genome. The comparison results were ideal.

**Table 2:** Summary table of sample data quality.

Sample name	clean_reads	clean_bases	error_rate	Q20	Q30	GC_pct	total_map	unique_map	multi_map
CF_PL_T1	40,586,638	6.09G	0.02	98.18	94.76	47.7	35,696,435 (87.95%)	32,773,321 (80.75%)	2,923,114 (7.2%)
CF_PL_T2	46,224,834	6.93G	0.02	98.21	94.85	47.79	41,197,358 (89.12%)	37,755,778 (81.68%)	3,441,580 (7.45%)
CF_PL_1	39,548,724	5.93G	0.02	98.16	94.67	47.93	34,605,233 (87.5%)	31,212,777 (78.92%)	3,392,456 (8.58%)
CF_PL_2	39,543,206	5.93G	0.02	98.16	94.72	47.96	34,473,806 (87.18%)	30,996,110 (78.39%)	3,477,696 (8.79%)
CF_RS_T1	43,555,680	6.53G	0.03	98.02	94.08	46.74	38,890,703 (89.29%)	36,036,140 (82.74%)	2,854,563 (6.55%)
CF_RS_T2	42,815,234	6.42G	0.02	98.13	94.39	46.74	38,510,147 (89.94%)	35,762,766 (83.53%)	2,747,381 (6.42%)
CF_RS_1	42,008,762	6.3G	0.02	98.31	94.98	46.97	37,539,271 (89.36%)	34,554,987 (82.26%)	2,984,284 (7.1%)
CF_RS_2	39,717,744	5.96G	0.02	98.21	94.7	47	35,319,209 (88.93%)	32,387,081 (81.54%)	2,932,128 (7.38%)
CF_DZ_T1	43,540,378	6.53G	0.02	98.06	94.22	47.64	29,847,868 (68.55%)	27,732,482 (63.69%)	2,115,386 (4.86%)
CF_DZ_T2	42,065,710	6.31G	0.02	98.11	94.31	47.18	32,408,006 (77.04%)	30,099,937 (71.55%)	2,308,069 (5.49%)
CF_DZ_1	39,302,374	5.9G	0.03	97.93	93.88	47.2	35,523,999 (90.39%)	32,751,905 (83.33%)	2,772,094 (7.05%)
CF_DZ_2	43,175,816	6.48G	0.02	98.05	94.19	47.01	38,943,394 (90.2%)	35,895,363 (83.14%)	3,048,031 (7.06%)
CF_RZ_T1	37,887,464	5.68G	0.02	98.04	94.28	48.19	29,142,172 (76.92%)	27,009,444 (71.29%)	2,132,728 (5.63%)
CF_RZ_T2	43,841,112	6.58G	0.02	98.06	94.32	47.68	36,527,776 (83.32%)	33,910,560 (77.35%)	2,617,216 (5.97%)
CF_RZ_1	40,618,892	6.09G	0.03	97.97	94.11	47.24	30,466,571 (75.01%)	27,551,816 (67.83%)	2,914,755 (7.18%)
CF_RZ_2	40,977,796	6.15G	0.02	98.14	94.73	47.08	30,427,392 (74.25%)	27,459,078 (67.01%)	2,968,314 (7.24%)
CF_TA_T1	40,416,324	6.06G	0.02	98.2	94.81	47.72	35,920,960 (88.88%)	32,686,412 (80.87%)	3,234,548 (8.0%)
CF_TA_T2	43,215,936	6.48G	0.03	97.68	93.37	47.77	37,798,309 (87.46%)	34,483,600 (79.79%)	3,314,709 (7.67%)
CF_TA_1	43,505,192	6.53G	0.02	98.09	94.35	46.78	37,830,821 (86.96%)	35,448,152 (81.48%)	2,382,669 (5.48%)
CF_TA_2	41,522,038	6.23G	0.03	97.78	93.53	46.86	35,807,199 (86.24%)	33,675,341 (81.1%)	2,131,858 (5.13%)

raw_reads, the number of reads in the original data; clean_reads, the number of reads after the original data was filtered; clean_bases, the number of bases after the original data was filtered; error_rate, the overall sequencing error rate of the data; Q20, the percentage of bases with a Phred value greater than 20 to the total bases; Q30, the percentage of bases with a Phred value greater than 30 to the total bases; GC_pct, the percentage of G and C in clean reads that account for the four bases; total_reads, The number of clean reads of sequencing data after quality control; total_map, the number and percentage of reads aligned to the genome; unique_map, the number and percentage of reads aligned to the unique position of the reference genome; multi_map, The number and percentage of reads aligned to multiple positions in the reference genome.

The number of expressed genes in the véraison stage to maturity stage of different areas was counted (Table 3). The results showed that the number of genes in the véraison period and maturein period were basically similar in each district, with more than 19,066. During the véraison stage, Rushan has a large number of genes, with 20,750; Tai'an has the lowest number of genes, only 19,066. At the mature stage, Tai'an has a large number of genes, with 20,735; and Rizhao has the lowest number of genes, only 19,176. Relatively speaking, the genes in the véraison period in Tai'an and Dezhou were less than in the mature period, which decreased by 1669 and 717 respectively. Penglai, Rushan and Rizhao had more genes in the véraison stage than in the mature stage, with an increase of 450, 326 and 1119 genes, respectively. Further using padj ≤ 0.05 and |log2FoldChange|≥ 0.0 as the threshold for screening DEGs, the analysis found that Tai'an has the largest number of DEGs expressed with 12,439, accounting for more than 59% of all expressed genes; the Penglai’s number of expressed genes was only 5386, accounting for more than 26% of all expressed genes.

**Table 3:** The number of expressed genes and the number of DEGs in each winery in different periods.

Area	The number of genes in the véraison stage	The number of genes in the maturity stage	The number of degs
PL	20,566	20,116	5386
RS	20,750	20,424	7823
DZ	19,571	20,288	8319
RZ	20,295	19,176	9695
TA	19,066	20,735	12,439

**Figure 3:**
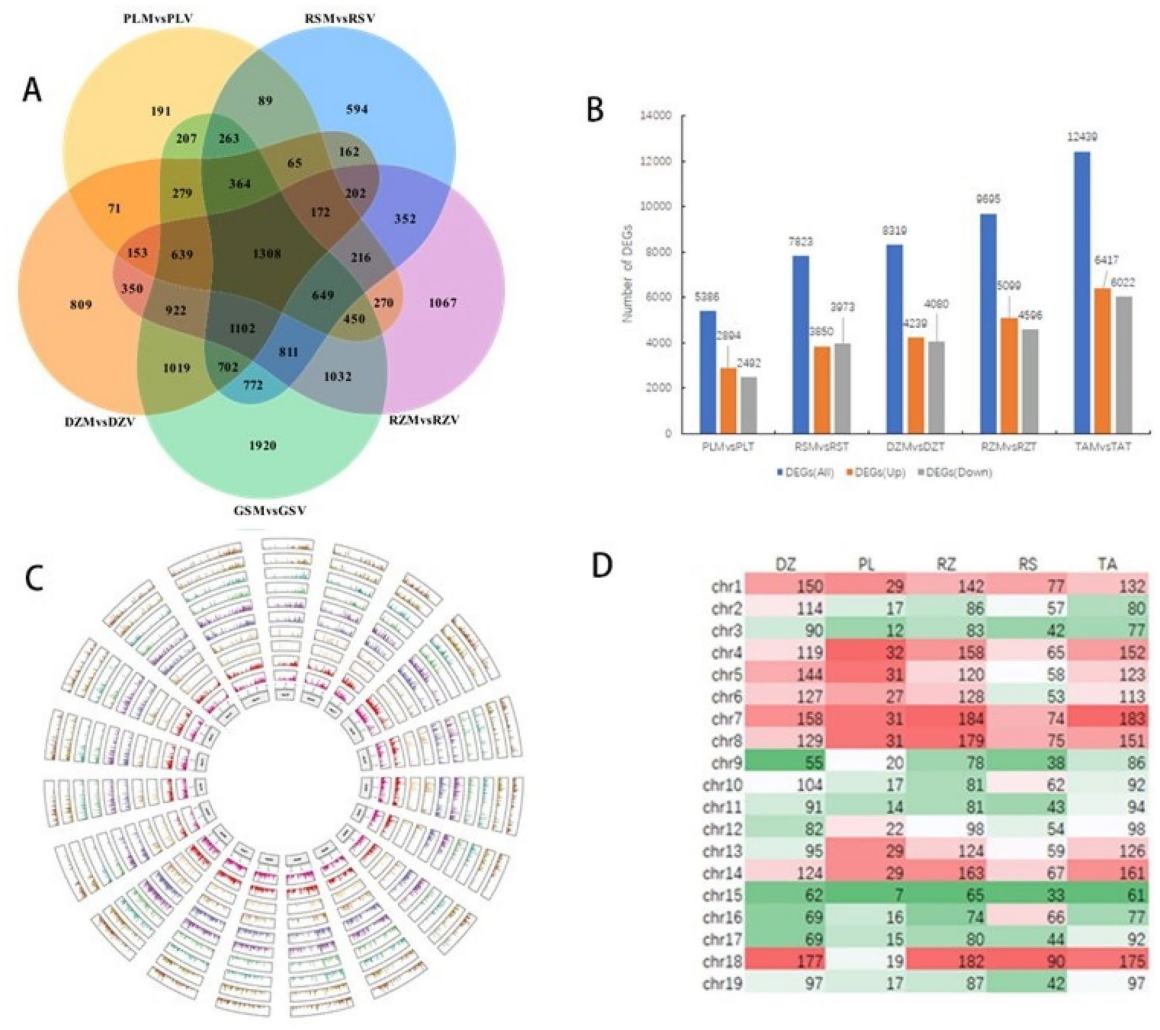
Analysis of DEGs in five different area. (**A**) Venn diagram of the comparison between the véraison period and the mature period in five area; (**B**) analysis of the number of DEGs up-regulated and down-regulated in five different area; (**C**) DEGs levels on different chromosomes in the five different area during the véraison stage and the maturity stage. Transcriptome data use maturity stage versus véraison stage. Log2FoldChange ≥ 1 and padj ≤ 0.05 as the screening conditions for significant expression levels. The circle graph uses the FPKM of the differential gene as a histogram, and takes log2FPKM to represent the expression level of all genes at the transcription level according to the average value of gene expression per 1000 bp according to the position of the gene on the chromosome. From the inside to the outside, the circles were Dezhou véraison stage (DZV)/Dezhou maturity stage (DZM)/Penglai véraison stage (PLV)/Penglai maturity stage (PLM)/Rizhao véraison stage (RZV)/Rizhao maturity stage (RZM)/Rushan véraison stage (RSV)/Rushan maturity stage (RSM)/Tai'an véraison stage (TAV)/Tai'an maturity stage (TAM). The heat map uses log2FoldChange to represent the expression level of all genes at the transcription level; D. transcriptome data use maturity stage versus véraison stage. Log2FoldChange ≥ 1 and padj ≤ 0.05 as the screening conditions for significant expression levels. Chromosomal distribution of differentially expressed genes in five wineries.

Among the 5 areas, Tai'an has largest number of DEGs with 12,439 (6417 up-regulated genes and 6022 down-regulated genes), while Penglai has the smallest number of DEGs with only 5386 (2894 genes up-regulated and 2492 genes down-regulated). The up-regulated and down-regulated genes of DEGs were further summarized and analyzed, and it was found that Rushan was the only area with more down-regulated genes than up-regulated genes, with a total of 7823 DEGs (3850 up-regulated genes and 3973 down-regulated genes) (Fig. 3B). Comparing the DEGs of ‘*Cabernet Franc*’ fruits in 5 areas, it was found that 1308 DEGs were the same in these areas. Interestingly, Tai'an area had 1920 genes different from other areas, and Penglai had only 191 (Fig. 3A). Use padj ≤ 0.05 and |log2FoldChange|≥ 1.0 as thresholds for screening DEGs with high expression levels (Fig. 3C). Using 0.5 Mb as the statistical interval, the locus distribution of expressed genes on 19 chromosomes was analyzed. The results showed that the number of highly expressed DEGs on chromosomes varied in different areas. The gene distribution of DEGs in Penglai was sparse on all chromosomes, and there were few differential genes with high expression. The change trend of highly expressed genes in the same area was similar in the véraison stage and the mature stage, but the number of genes distributed on each chromosome was inconsistent. The denser the gene distribution on the chromosome, the more highly expressed DEGs. The study found that Dezhou has the most DEGs on chromosome 18, and the least on chromosome 9; Penglai has more and evenly distribution on chromosomes 1, 4–8, 13, and 14, and the least distribution on chromosome 15; Rizhao and Tai'an have the most DEGs distributed on chromosome 7. The most DEGs were distributed on chromosome 18 in Rushan, and the least distributed on chromosome 15. On the whole, chromosome 7 has the most DEGs in each area, and chromosome 15 has the least DEGs in each area (Fig. 3D).

### DEGs analysis of GO and KEGG in the véraison and maturity period of ‘*Cabernet Franc*’ in different area

**Figure 4:**
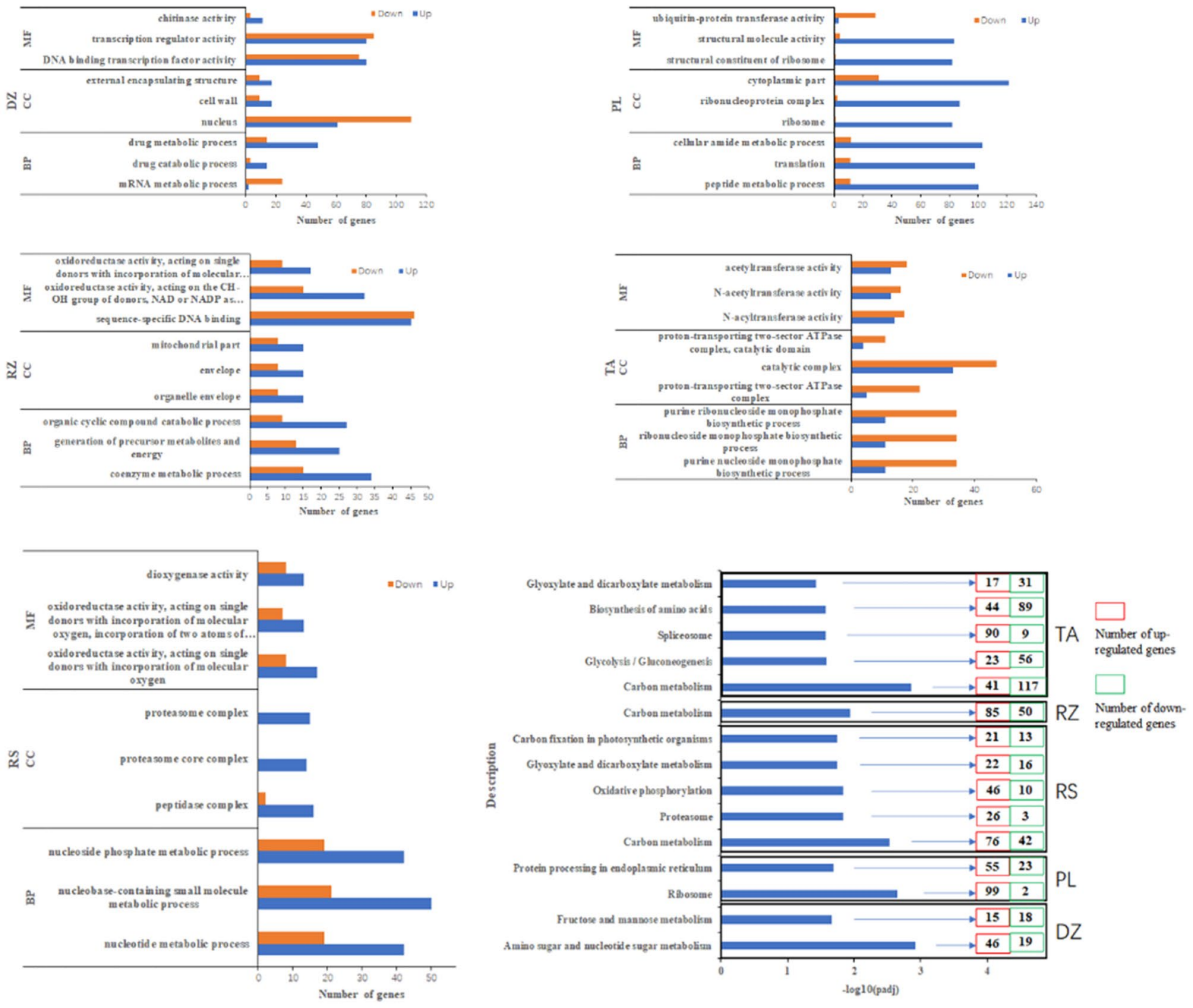
Differential gene GO (functional classification) and KEGG (metabolic pathway) enrichment analysis in five different area. In GO enrichment analysis, blue represents the number of down-regulated genes; red represents the number of up-regulated genes. In the KEGG analysis, blue represents the total number of DEGs, the red box represents the number of up-regulated genes, and the green box represents the number of down-regulated genes.

Gene Ontology (GO) analysis was divided into three parts: MF (Molecular Function), BP (biological process) and CC (cellular component). Using the GOseq method in Blast2GO for analysis, GO terms with padj < 0.05 were considered to be significantly richer. The study found that a total of 855 GO terms were involved in the 5 areas, including 444 BPs, 113 CCs, and 296 MFs. The top 3 most abundant GO term entries in each section by area were displayed (Fig. 4). The top 3 richest GO terms were found to be different in each area, among them, the GO terms of DZ, RZ, RS, and PL have more up-regulated genes, and TA has more down-regulated genes. “Oxidoreductase activity, acting on single donors with incorporation of molecular oxygen” was abundant in both Rushan and Rizhao MFs.

In each area, the first 5 channels with padj ≤ 0.5 were selected for KEGG analysis. The study found that 116 KEGG pathways were annotated in 5 areas. Among them, Dezhou's “amino sugar and nucleotide sugar metabolism”, “fructose and mannose metabolism” pathways were significant; Penglai's “ribosome”, “protein processing in endoplasmic reticulum” pathways were significant; Rushan's “carbon metabolism”, “proteasome”, “oxidative phosphorylation”, “glyoxylate and dicarboxylate metabolism”, “carbon fixation in photosynthetic organisms” pathways were significant; Rizhao's “carbon metabolism” pathway was significant; Tai’an's “carbon metabolism”, “glycolysis/gluconeogenesis”, “spliceosome”, “biosynthesis of amino acids”, “glyoxylate and dicarboxylate metabolism” pathways were significant. It was worth noting that Rushan, Rizhao, and Tai'an all have “carbon metabolism”. Carbon metabolism was the most fundamental aspect of life. Carbon metabolism pathways including glycolysis, pentose phosphate pathway, and citric acid cycle, as well as 6 known carbon fixation pathways and some methane metabolism pathways. “Carbon metabolism” in Tai'an has 41 up-regulated genes and 117 down-regulated genes. But Rizhao was regulated by 85 up-regulated genes and 50 down-regulated genes, with more up-regulated genes than down-regulated genes. Rushan also has more up-regulated genes than down-regulated genes that regulate carbon metabolism, indicating that the degree of carbon metabolism was different in different area. Rushan and Tai'an both have “glyoxylate and dicarboxylate metabolism”. Tai'an has more up-regulated genes, and Rushan was the opposite.

### Analysis of key genes for sugar and anthocyanin content in five areas

The sugar content and anthocyanin content of grapes were one of the important qualities in winemaking. We found that '*Cabernet Franc*' in Tai'an was rich in soluble sugar, glucose and fructose, while it was the opposite from Dezhou (Fig. 2A). Transcriptome data show that the number of up-regulated DEGs in “sucrose metabolic process” and “sucrose synthase activity” in Tai'an was higher than that in Dezhou, and the number of down-regulated DEGs was lower than that in Dezhou. In the "Fructose and mannose metabolism" pathway, Tai'an has 13 up-regulated genes and 25 down-regulated genes, while Dezhou has 15 up-regulated genes and 8 down-regulated genes (Table 4).The “Flavonoid biosynthesis” was an important pathway for the synthesis of anthocyanins, anthocyanins were a very important class of polyphenols in grapes. Rushan had 20 up-regulated genes and 5 down-regulated genes, while Tai'an had 8 up-regulated genes and 28 down-regulated genes.

**Table 4:** Some trait-related metabolic pathways and number of DEGs in transcriptome data.

Type	Name	Dezhou		Rushan		Rizhao		Tai’an		Penglai	
		Up	Down	Up	Down	Up	Down	Up	Down	Up	Down
Sugar	Sucrose metabolic process	2	4	3	0	3	2	4	3	1	2
	Sucrose synthase activity	1	4	1	0	2	2	4	2	0	2
	Fructose and mannose metabolism	15	8	14	13	19	14	13	25	9	7
Acid	Citrate cycle (tca cycle)	16	7	21	3	23	6	7	28	13	8
	Ascorbate and aldarate metabolism	17	11	9	10	9	20	11	23	10	8
Anthocyanins	Flavonoid biosynthesis	11	11	20	5	19	9	8	28	5	14

**Figure 5:**
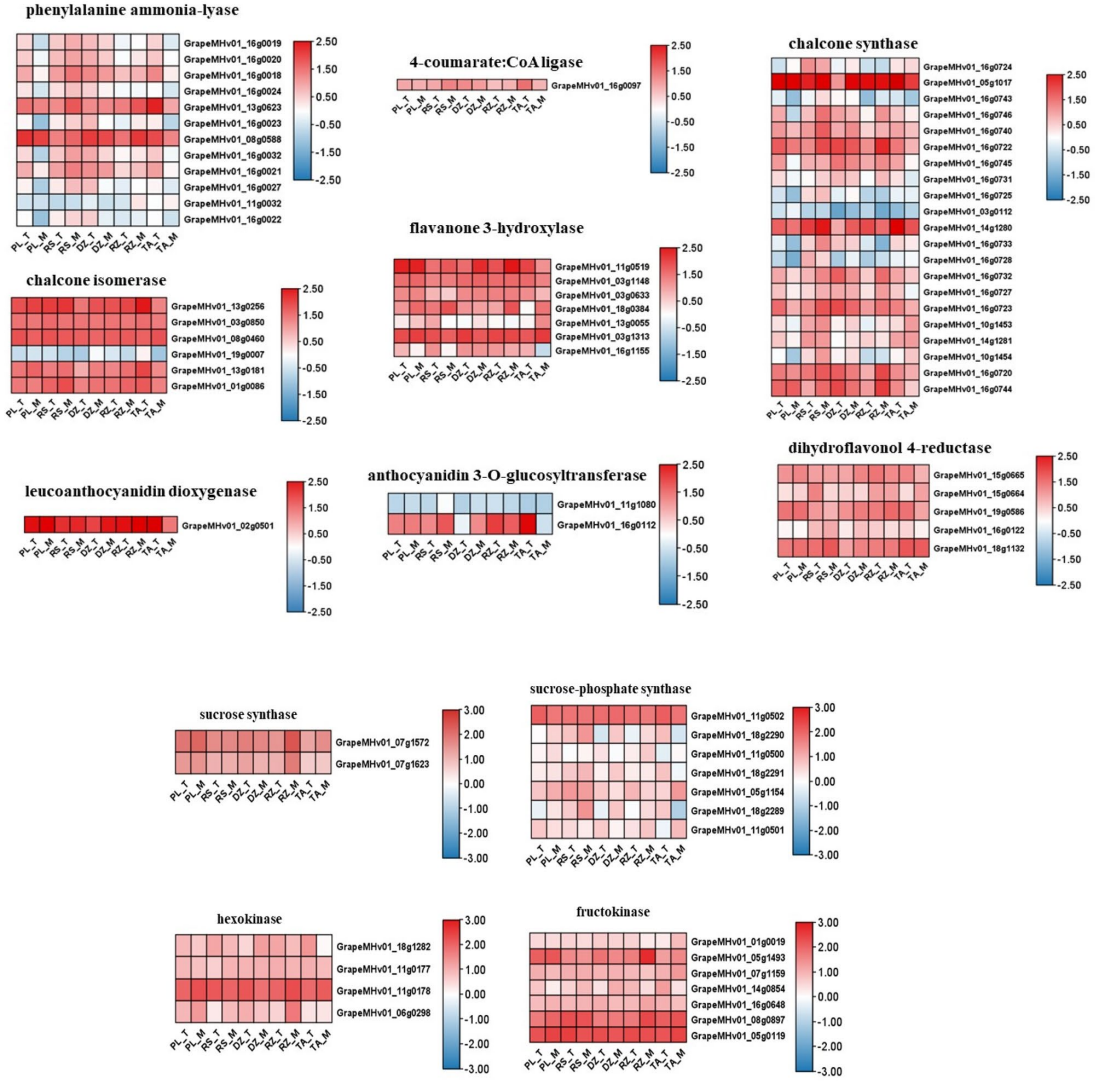
Heatmap of genes related to sugar and anthocyanin biosynthesis in ‘*Cabernet Franc*’ at veraison and maturity in five areas.

RNA-seq was used to evaluate the transcript levels of genes related to ‘*Cabernet Franc*’ and anthocyanin synthesis pathways in five areas (Fig. 5). Enzymes involved in the phenylpropanoid metabolic pathway include phenylalanine ammonia-lyase, cinnamate-4-hydroxylase and 4-coumarate: CoA ligase, among which GrapeMHv01_13g0623 and GrapeMHv01_08g0588 are important genes encoding phenylalanine ammonia-lyase, GrapeMHv01_16g0097 is the main gene encoding 4-coumarate: CoA ligase. Enzymes involved in the flavonoid pathway mainly include chalcone synthase, chalcone isomerase, flavanone 3-hydroxylase, dihydroflavonol 4-reductase, leucoanthocyanidin dioxygenase, anthocyanidin 3-O-glucosyltransferase. Among them, GrapeMHv01_05g1017 is an important gene encoding chalcone synthase, GrapeMHv01_13g0256 and GrapeMHv01_08g0460 is a key gene encoding chalcone isomerase, GrapeMHv01_11g0519 and GrapeMHv01_03g1313 is a key gene encoding flavanone 3-hydroxylase, GrapeMHv01_19g0 586 and GrapeMHv01_18g1132 is the key gene encoding dihydroflavonol 4-reductase, GrapeMHv01_02g0501 is the key gene encoding leucoanthocyanidin dioxygenase, GrapeMHv01_16g0112 is the key gene encoding anthocyanidin 3-O-glucosyltransferase. Surprisingly, most of the genes regulating phenylalanine ammonia-lyase and chalcone synthase are located on chromosome 16. At the same time, comparing the expression levels of key genes regulating anthocyanin synthesis in the five areas during the maturity period, it was found that the expression levels of most key genes regulating anthocyanin synthesis in the DZ area were lower than other areas, which is consistent with the trend of anthocyanin content. Comparing GrapeMHv01_05g1017 and GrapeMHv01_11g0519 with the highest expression during anthocyanin synthesis, it was found that the trends of individual genes in the five regions were not the same, but the trend of GrapeMHv01_05g1017 was similar to the anthocyanin content trend we measured.

Sucrose synthase, sucrose-phosphate synthase, hexokinase, and fructokinase are key enzymes in grapes that regulate sucrose metabolism and fructose formation. Among them, GrapeMHv01_07g1572 is obviously the key gene that regulates sucrose synthase, GrapeMHv01_11g0502 is the key gene that regulates sucrose-phosphate synthase, GrapeMHv01_11g0178 is the key gene that regulates hexokinase, GrapeMHv01_05g1493, GrapeMHv01_08g0897 and GrapeMHv01_05g0119 is a key gene that regulates fructokinase. At the same time, comparing genes related to regulating sucrose metabolism in the five areas during the maturity period, it was found that the expression levels of most genes in the RZ area were higher than other areas, the expression levels of most genes in the DZ region are lower than those in other regions, which is similar to the trend of the soluble sugar content we measured. Surprisingly, the trends of GrapeMHv01_08g0897 were similar to the trends of soluble sugar content in the five areas.

## Discussion

**Figure 6:**
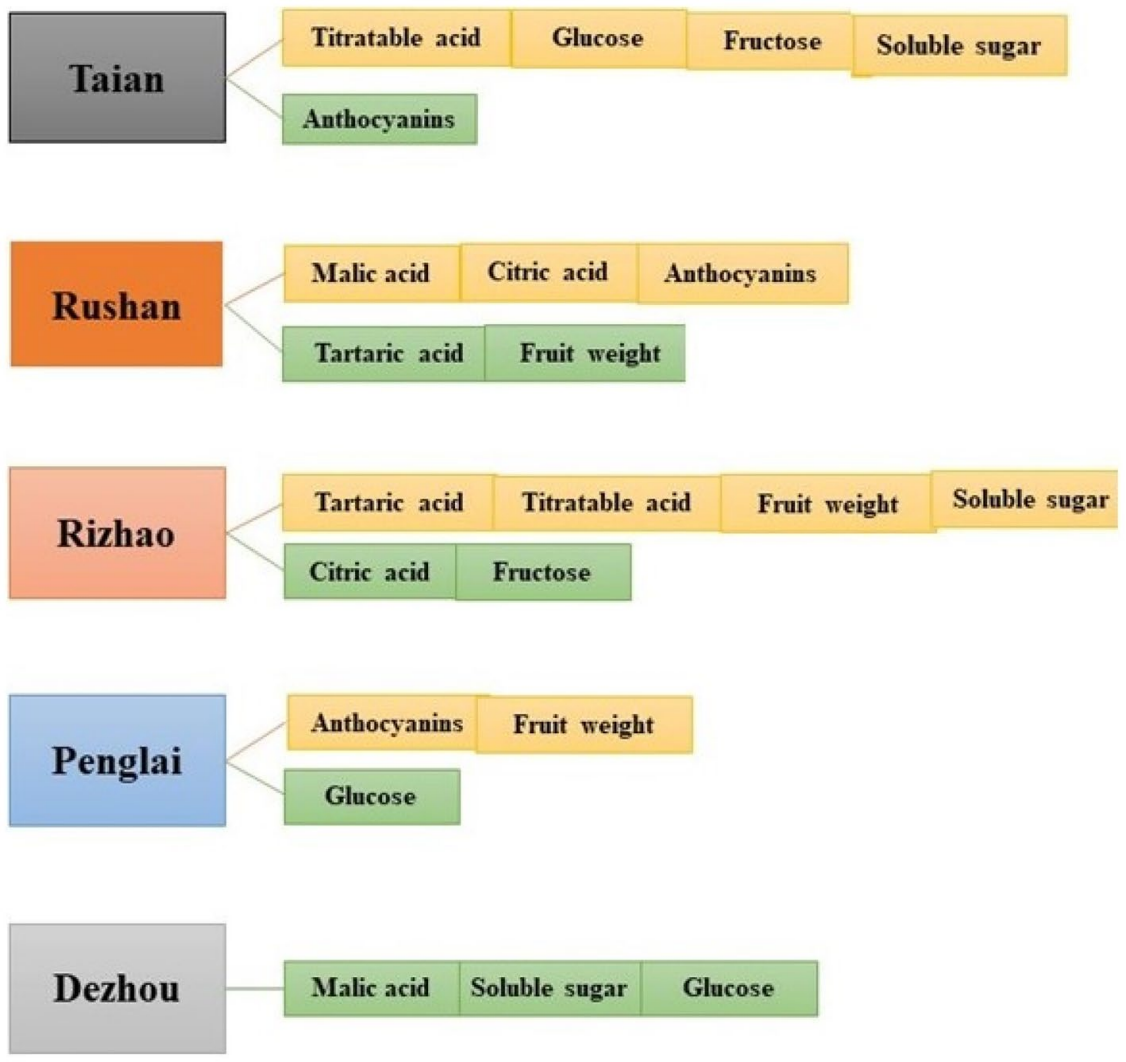
Agronomic characteristics of ‘*Cabernet Franc*’ in different areas. Yellow means the area's ‘*Cabernet Franc*’ is rich in the substance, green means the area's *Cabernet Franc* is low in the substance.

Shandong is one of the main wine producing areas in China. Due to the diverse topography of Shandong producing areas, the climatic environment of each area is also different, resulting in different quality of ‘*Cabernet Franc*’ grapes. In Shandong province, the quality of ‘*Cabernet Franc*’ varies greatly from area to area. Different geographic locations had a greater impact on agronomic traits (Fig. 6).

In conclusion, compared with other areas, the Rizhao area was rich in soluble sugar, and had larger single fruit weight and fruit volume. Tai'an area is rich in titratable acid content. Relatively speaking, among the agronomic traits of the five areas, Rizhao and Tai'an were at the upper-middle level except for anthocyanin content. Since Rizhao is 146 m above sea level, Tai'an is 193 m above sea level, Dezhou is 25 m above sea level, Rushan is 27 m above sea level, Penglai is 67 m above sea level, therefore, it indicated that altitude had a higher effect on soluble sugar, titratable acid, fruit volume and weight. For anthocyanins, the content of anthocyanins increased from south to north, indicating that the content of anthocyanins was related to geographical location. It was preliminarily speculated that the reason for the difference in anthocyanin content in the two areas was due to the north–south climate. Relatively speaking, the temperature in the south of Shandong is high and rainy, while the temperature in the north is low and little rainy. Low temperature contributed to the accumulation of anthocyanins^19^. Another reason for this situation may be that compared with latitude, the height difference of about 100 m has less effect on anthocyanins. Even though previous research has shown that anthocyanins increase with altitude^20,21^.

For sugar and acid components, there are differences in the content of sugar and acid components in different areas. Among them, the glucose and fructose contents in Dezhou were low, only 137.14 mg/g and 98.51 mg/g, but the glucose and fructose contents in Tai'an were rich, which were 248.4 mg/g and 273.32 mg/g, respectively. We tentatively concluded that this may be due to altitude. Because both Tai'an and Dezhou are located in the interior of Shandong, and the altitude of Tai'an is 193 m, while that of Dezhou is 25 m. Previous studies showed that high altitude can increase the content of sugar^22,23^. This is similar to our research. For the acid component, the main components of the acid are different in different areas. Penglai, Dezhou, Rizhao and Tai'an are all dominated by tartaric acid, while Rushan is dominated by malic acid. Grapes are rich in tartaric acid, but the synthesis pathway of tartaric acid is not yet clear. Both sugar content and anthocyanin content are affected by environmental factors such as altitude, temperature, moisture, etc^24,25^. High temperature can increase photosynthesis, promote sugar biosynthesis and transport^22^. Among the five areas, Rizhao had the highest tartaric acid content, and Rushan had the lowest tartaric acid content, which may be due to the fact that Rizhao is located in the south of Shandong, while Rushan is in the north of Shandong, and the altitude of Rizhao is higher than that of Rushan. Therefore, we speculate that the altitude and latitude is related to the content of tartaric acid, but the specific mechanism of action needs further study.

From the véraison stage to the maturity stage, the longitudinal diameter, transverse diameter and titratable acid of ‘*Cabernet Franc*’ in the five areas did not change much, while anthocyanin and single fruit weight changed greatly. It may be because the fruit has passed the expansion period, so the longitudinal and transverse diameters have not changed much. And anthocyanins increase with the coloration of the fruit. During the process from véraison stage to maturity stage, the degree of change of characters in different areas was inconsistent. The anthocyanins in Penglai and Rushan vary greatly, which may be due to the fact that Rushan is located in the south of Shandong province, near the sea, and the temperature is relatively low, which is favorable for fruit coloring.

Transcriptome analysis found that in most areas, there were more differential genes at the véraison stage than maturity stage, which indicated that the fruit was metabolized during the turning stage. Further analysis found that the number of DEGs and up-regulated genes was different in different areas. There are 12,439 DEGs in Tai'an (5417 up, 6022 down); 9,695 DEGs in Rizhao (5099 up; 4596 down); 8,319 DEGs in Dezhou (4239 up, 4080 down); 7,823 DEGs in Rushan (up 3850, down 3973); Penglai has 5386 DEGs (up 2894; down 2492), the number of DEGs is associated with the superiority of agronomic traits, and the areas with more superiority also have more DEGs. From the physiological data, and Rizhao had the best agronomic traits, which corresponded to the number of DEGs. On the whole, chromosome 7 has the most DEGs in each area, and chromosome 15 has the least DEGs in each area. It is preliminarily speculated that geographical location may regulate the quality traits of grapes mainly by affecting the genes on chromosome 7 of grapes. Of course, the specific mechanism of action needs further study.

The study found that DEGs in the five areas annotated a total of 855 GO items and 166 KEGG pathways. Among them, the analysis of GO projects and KEGG pathways related to agronomic traits found that the number of up-regulated/down-regulated genes may be related to the agronomic traits exhibited by fruits. The study found that the expression levels of most key genes regulating anthocyanin synthesis in the DZ area were lower than other areas, which was consistent with the results of trait studies, and the expression trend of GrapeMHv01_05g1017 in the five areas was similar to the trend of anthocyanin content. GrapeMHv01_05g1017 is a key gene that regulates chalcone synthase, indicating that compared with other enzymes, chalcone synthase plays an important role in the synthesis of anthocyanins in '*Cabernet Franc*'. And the expression levels of most genes regulating sugar synthesis in the RZ area were higher than other areas, the expression levels of most genes in the DZ region are lower than those in other regions, which is similar to the trend of the soluble sugar content we measured. The trends of GrapeMHv01_08g0897 was similar to the trends of soluble sugar content in the five areas. GrapeMHv01_08g0897 It is a key gene that regulates fructokinase, indicating that fructokinase may be a key enzyme affecting sugar synthesis. We speculate that gene expression and trait measurement data are related to each other, but it is unreliable to judge fruit quality based solely on gene expression. Gene expression is only suitable for judging samples with large differences in quality, and comparing them to determine the highest or lowest quality. However, if the quality difference between samples is small, it is a wrong decision to characterize the quality based on gene expression.

And alpha-amylase, sucrose synthase, and hexokinase related to fructose-glucose biosynthesis and metabolism, RNA-seq found that Tai'an had the most up-regulated genes, this is the same trend as the levels of glucose and fructose in the 5 areas. Malic acid, citric acid, and tartaric acid are the main reasons for the sour taste of fruit juice^13^. Compared with sugar, the content of titratable acids in fruits is lower, but titratable acids have an important impact on fruit quality. The biosynthesis of malic acid is mainly derived from the tricarboxylic acid cycle (TCA) pathway. Malate metabolism in fruit is mainly through phosphoenolpyruvate (PEP) under the action of phosphoenolpyruvate carboxylase (PEPC), which catalyzes the formation of oxaloacetate (OAA) and inorganic phosphate from PEP and CO_2_. OAA is reduced to malate by NAD-malate dehydrogenase (NAD-MDH)^26^. Citrate synthase, phosphoenolpyruvate carboxylase, and aconitase are involved in citrate synthesis, and they are regulated by GrapeMHv01_12g0317/GrapeMHv01_12g0071/GrapeMHv01_12g0640, respectively. During the maturity stage, the expression levels of these three genes in Rushan were all high. Wine tartaric acid, the L ( +) isomer, is a strong acid that directly interferes with the pH of wine and has antioxidant effects^13^. Tartaric acid is synthesized from L-ascorbic acid, however, the up-regulated genes of ascorbate and aldarate metabolism in Penglai are more than those in Rushan. In conclusion, by studying the agronomic traits of '*Cabernet Franc*' in different areas of Shandong province, we found that the same variety has different agronomic traits in different places, and its transcriptome data also verified this view. This study helps to deepen the understanding of the regional quality of grapes, provides a new theoretical basis for the regional analysis of the quality of '*Cabernet Franc*' grape berries, provides a reference for the selection of an excellent geographical environment for the cultivation of '*Cabernet Franc*', and lay the foundation for construction of the gene regulation network of ‘*Cabernet Franc*’ affected by geographical location.

## Data Availability

All data generated or analysed during this study are included in this published article. The datasets presented in this study can be found in online repositories. The names of the repository/repositories and accession number (s) can be found at: NCBI Accession (PRJNA932956) and accessions SRR23502979-23,502,998.
