# Author Correction: Geographical location influence ‘*Cabernet Franc*’ fruit quality in Shandong province

**DOI:** 10.1038/s41598-024-58397-w

**Published:** 2024-04-04

**Authors:** Chaoping Wang, Xueqin Chen, Yanhua Ren, Xuxian Xuan, Tariq Pervaiz, Lingfei Shangguan, Jinggui Fang

**Affiliations:** 1 College of Horticulture, Nanjing Agricultural University, Nanjing, Jiangsu Province China; 2 Shandong Academy of Grape, Jinan, Shandong Province China

Correction to: *Scientific Reports* 10.1038/s41598-023-50140-1, published online 29 January 2024

The original version of this Article omitted an affiliation for Chaoping Wang. The correct affiliations are listed below.

College of Horticulture, Nanjing Agricultural University, Nanjing, Jiangsu Province, China.

Shandong Academy of Grape, Jinan, Shandong Province, China.

In addition, the Funding section contained an error.

“This research was funded by Shandong Provincial Key Research and Development Plan (Agricultural Improvement Project) 2022LZGCQY1018, General Project of National Natural Science Foundation of China (32272647), National Key Research and Development Project 2019YFD1000103, National Key Research and Development Project 2019YFD1002500.”

now reads:

“This research was funded by Shandong Provincial Key Research and Development Plan (Agricultural Improvement Project)2022LZGCQY1018, General Project of National Natural Science Foundation of China (32272647), National Key Research and Development Project 2019YFD1000103, National Key Research and Development Project 2019YFD1002500, and Collaborative Promotion Plan of Major Agricultural Technologies in Shandong Province in 2023-Integrated Demonstration and Promotion of Key Technologies for Grape Quality Improvement and Efficiency-enhancing green Production (SDNYXTTG-2023-18)."

The original Article has been corrected.

